# *Streptomyces* spp. From Ethiopia Producing Antimicrobial Compounds: Characterization via Bioassays, Genome Analyses, and Mass Spectrometry

**DOI:** 10.3389/fmicb.2018.01270

**Published:** 2018-06-12

**Authors:** Moges Kibret, Jaime F. Guerrero-Garzón, Ernst Urban, Martin Zehl, Valerie-Katharina Wronski, Christian Rückert, Tobias Busche, Jörn Kalinowski, Judith M. Rollinger, Dawit Abate, Sergey B. Zotchev

**Affiliations:** ^1^Microbial, Cellular and Molecular Biology Department, College of Natural Science, Addis Ababa University, Addis Ababa, Ethiopia; ^2^Department of Pharmacognosy, University of Vienna, Vienna, Austria; ^3^Department of Pharmaceutical Chemistry, University of Vienna, Vienna, Austria; ^4^Department of Analytical Chemistry, Faculty of Chemistry, University of Vienna, Vienna, Austria; ^5^Center for Biotechnology, Bielefeld University, Bielefeld, Germany

**Keywords:** ethiopian soils, *Streptomyces*, solid state fermentation, antimicrobial assays, high resolution mass spectrometry, NMR-assisted structure elucidation, genome analyses

## Abstract

A total of 416 actinomycete cultures were isolated from various unique environments in Ethiopia and tested for bioactivity. Six isolates with pronounced antimicrobial activity were chosen for taxonomic identification and further investigation. Morphological and cultural properties of the isolates were found to be consistent with those of the genus *Streptomyces*, which was further confirmed by phylogenetic analysis based on 16S rRNA gene sequences. One of the isolates, designated *Streptomyces* sp. Go-475, which displayed potent activity against both pathogenic yeasts and Gram-positive bacteria, was chosen for further investigation. Metabolite profiles and bioactivity of Go-475 incubated on wheat bran-based solid and soya flour-based liquid media were compared using high-resolution LC-MS. This allowed identification of several known compounds, and suggested the ability of Go-475 to produce new secondary metabolites. Major anti-bacterial compounds were purified from liquid cultures of Go-475, and their structures elucidated by NMR and HRMS as 8-O-methyltetrangomycin and 8-O-methyltetrangulol. In addition, many potentially novel metabolites were detected, the majority of which were produced in solid media-based fermentation. The genome sequence of *Streptomyces* sp. Go-475 was obtained using a hybrid assembly approach of high quality Illumina short read and low quality Oxford Nanopore long read data. The complete linear chromosome of 8,570,609 bp, featuring a G+C content of 71.96%, contains 7,571 predicted coding sequences, 83 t(m)RNA genes, and six *rrn* operons. Analysis of the genome for secondary metabolite biosynthesis gene clusters further confirmed potential of this isolate to synthesize chemically diverse natural products, and allowed to connect certain clusters with experimentally confirmed molecules.

## Introduction

Actinomycete bacteria have a widely recognized potential for the production of bioactive secondary metabolites and valuable enzymes (van der Meij et al., 2017). These Gram-positive bacteria belonging to the order *Actinomycetales* are abundant in soils, marine sediments as well as being associated with various plants and animals (Goodfellow and Williams, 1983). Most importantly, secondary metabolites of actinomycetes play a prominent role in the history of drug development, in particular as antibiotics and anticancer agents (Katz and Baltz, 2016). During the golden age of natural product-based drug discovery (1950s through 1970s), most of the antibiotics currently used to treat various infections were discovered from actinomycetes; e.g., the antifungals nystatin and amphotericin or the antibacterials erythromycin and vancomycin. Besides antibiotics, actinomycetes are known to produce antitumor agents (Li et al., 2008), immunosuppressants, pesticides, herbicides, and anthelmintics (El-Tarabily et al., 2009; Liu et al., 2009; Nakouti et al., 2012). Actinomycetes of the genus *Streptomyces* are the most versatile producers of bioactive secondary metabolites and they continue to be interesting sources for the discovery of new antibiotics (Berdy, 2005; Baltz, 2007; Demain, 2009). Streptomycetes usually possess 20–50 gene clusters dedicated to the biosynthesis of secondary metabolites in their genomes, underscoring their potential for the discovery of new bioactive compounds (Nett et al., 2009). Most of such clusters remain “silent” in laboratory conditions, but tools are being developed for activation of these genes, thus prompting production of previously undetected compounds (Kealey et al., 2017).

Despite the onset of the genomic era and some progress in awakening “silent” gene clusters in actinomycetes, screening for new natural products remains an important part in the drug discovery process. The major challenge in this approach remains the re-discovery of already known bioactive compounds, even from phylogenetically distinct actinomycetes. Therefore, efficient de-replication (Carrano and Marinelli, 2015), novel screening techniques, and investigating new sample sources from underexplored habitats (Nolan and Cross, 1988; Tan et al., 2009; Goodfellow et al., 2010) become more and more important. In search for actinomycetes capable of producing novel bioactive metabolites, it appears especially beneficial to look into extreme habitats and unique environmental niches (Sanglier et al., 1996; Bull and Stach, 2007; Berdy, 2012; Matsui et al., 2012; Zotchev, 2012). In this regard, Ethiopian soils from various climatic zones possessing remarkable biodiversity may harbor new actinomycete strains that have the potential for new antibiotic discovery.

The current work was aimed at the isolation of antibiotic-producing actinomycetes from soils collected at various unique environments and specific biotopes in Ethiopia, with Ataye, Dengego, Gode, and Menagesha yielding bioactive isolates that were closely investigated in this study. Map of the region and the description of collection sites are provided in Figure S1 and its legend (Supplementary Materials). Standard screening methods were employed for initial detection of antimicrobial activities of the isolates, targeting known microbial pathogens. This was followed by taxonomic identification of the most active isolates and their bioactivity profiling. The genome of one isolate was sequenced, analyzed for the presence of secondary metabolite biosynthesis gene clusters (BGCs), and secondary metabolite profiles of its extracts were investigated using high-resolution MS. Both the genome and the metabolite profiles suggest potential for production of many presumably novel compounds. Identities of two known compounds were confirmed via isolation and NMR-assisted structure elucidation, and gene cluster for their biosynthesis was identified.

## Materials and methods

### Soil sampling and isolation of actinomycetes

The sample collection sites encompassed 13 specific habitats in five geographical regions, with diverse natural environments. Specific soil sampling locations and their corresponding coordinates are presented in Table S1 (Supplementary Materials). Soil samples were collected using sterile spatula by removing 2–3 inches of top soil and placing the material in sterile zipped polythene bags (Bharti et al., 2010). Sample bags were labeled according to their source and collection time. The collected soil samples were transported to the Microbiology Laboratory (Addis Ababa University) for isolation of bacteria.

Actinomycete cultures were isolated by soil serial dilution plating technique on various media including starch casein agar, starch nitrate agar, yeast malt extract agar, and actinomycete isolation agar medium supplemented with 50 μg/ml cycloheximide and 25 μg/ml nystatin. The cultures were incubated at 30 ± 2°C for 7–12 days. Observation was made daily during incubation period for colony growth (Okazaki et al., 1983; Hayakawa and Nonomura, 1987; Seong et al., 2001). Morphologically identified colonies and those showing a characteristic inhibition (clear zone) in the crowded plate were picked up with sterile wire loop and streaked on starch casein agar medium, yeast malt extract agar medium plates, and actinomycete isolation agar medium and incubated at 30°C for 7–12 days. Repeated re-streaking was conducted for further purification of the cultures. Pure actinomycete cultures were stored at 4°C on starch casein agar slants for further work.

### Taxonomic identification of selected isolates

Morphological and cultural characteristics of the isolates were determined as per the international streptomyces project guidelines (Shirling and Gottlieb, 1966). Micro-morphological structures of the isolates were studied as described in Williams and Cross (1971) and Williams et al. (1989) using a cover slip technique. The structure of the hyphae and the spore chain morphology were investigated using a light microscope (model BX-51; Olympus, Tokyo, Japan).

Identification of actinomycetes to the genus level was done by a polyphasic approach, including cultural, morphological, physiological, and biochemical characteristics as described in Basilio et al. (2003). Taxonomic identification of the isolates was also verified by the 16S rRNA gene sequence analysis. Genomic DNA of the isolates grown in 3% TSB medium (24–48 h, 28°C, 200 rpm) was extracted with DNeasy Tissue Kit (QIAGEN) following the instructions from the manufacturer. PCR amplification of the 16S rDNA fragments was performed using 27f and 1492r primers designed by Weisburg et al. (1991), and PCR products were directly sequenced at Eurofins Scientific (Austria) using the same primers. Phylogeny was inferred using the software MEGA7 (Kumar et al., 2016). Nucleotide sequences were deposited to the GenBank under accession numbers as follows: MF474325 (Ac-146), MF474326 (Go-475), MF474327 (Ac-123), MF474328 (Ed-065), MF474329 (Ru-355), MF474330 (Ac-006).

### Solid and liquid state fermentations and preparation of extracts

The inoculum for each of the selected isolates was prepared by inoculating three loopfulls of the spores in 250 mL Erlenmeyer flasks containing 50 mL of liquid ISP2 medium and incubating at 30°C on a rotary shaker (200 rpm) for 7 days. On the seventh day the culture was transferred to sterile test tubes and centrifuged at 2,000 rpm. The supernatant was discarded and the culture pellet was resuspended in 0.1 M phosphate buffer (pH 7.0) and 0.01% of sterile Tween 80 was added. The suspension prepared in such a way was used as an inoculum for solid state fermentation (SSF) experiments. For the SSF, 10 g of the solid substrate (wheat bran) was placed in 250 mL Erlenmeyer flasks and supplemented with 15.75 mL mineral salt solution containing 0.1% NaCl, 0.1% MgSO_4_, 0.5% NH_4_NO_3_, and 0.2% KH_2_PO_4_ supplemented with 1% each of soluble starch and peptone to produce moisture content of 65%. One milliliter of a trace elements solution containing (g/l): ${\text{CuSO}}_{4}^{.}$5H_2_O, 3.3; ${\text{FeSO}}_{4}^{.}$7H_2_O, 10.0; ${\text{ZnSO}}_{4}^{.}$7H_2_O, 50.0; ${\text{MnSO}}_{4}^{.}$2H_2_O, 4.0; CoCl_2_, 2.0; (NH_4_)_2_MoO_4_, 1.0, was added. Three mL of inoculum suspensions (containing 2.8 × 10^6^ CFU/mL) was added, shacked well to distribute the inoculum uniformly and incubated at 30°C for 12 days (Bussari et al., 2008). At the end of the fermentation process, methanol extracts were prepared as described in Venkateshwarlu et al. (2000).

For the liquid state fermentations (LSF), 15 mL of TSB medium (Oxoid, UK) was inoculated with 200 μL of spore suspension of isolate Go-475 in a 100 mL Erlenmeyer flask and incubated at 28°C with 200 rpm for 48 h to produce seed culture. The mycelium was collected by centrifugation and then resuspended in 10 mL sterile 20% glycerol solution for preparation of stocks. Two-hundred and fifty milliliters of medium 5288 (Soy flour 10 g, Glycerol 15 g, NaCl 5 g, CaCO_3_ 1 g, ${\text{CoCl}}_{2}^{.}$7H_2_O 1 mg, in 1 L of distilled water according to Wink, 2012) were inoculated with 5 mL of seed culture stock and fermented in 2 L baffled Erlenmeyer flasks at 28°C with 200 rpm for 7 days. The whole culture was freeze-dried and extracted with 50 mL of methanol.

### Antimicrobial activity testing

Primary screening for antifungal activity was performed for all pure actinomycete cultures using the agar plug method (Bharti et al., 2010) with *Candida albicans* ATCC 62376 and *Cryptococcus neoformans* (clinical isolate) as test organisms. The experiments were conducted in triplicates and the bioactivity of the isolates was assessed by the formation of an inhibition zone around the plug.

The bioactivity testing of the methanol extracts obtained from the isolates selected based on primary screening results was done for *C. albicans* and *C. neoformans* using the disc diffusion assay method (Galvan et al., 2008). Disc diffusion assay method described in CLSI (2012) was used for antibacterial susceptibility testing with the test organisms *Staphylococcus aureus* ATCC 25923, *Bacillus subtilis* ATCC 6633, *Escherichia coli* ATCC 25922, *Salmonella typhimurium* ATCC 6539, and *Shigella boydii* (clinical isolate). Description of the clinical isolates used as test organisms in antimicrobial assays is given in Supplementary Materials. All the secondary bioactivity tests with crude extracts were conducted in triplicates and inhibition zone diameters were measured. The data were analyzed by one- way- ANOVA and the mean separation was achieved by the Duncan's multiple range tests, the mean ±SD was used for interpretation of the data, using SPSS (version 20). Numerical differences were considered as significant at the probability level of *p* ≤ 0.05.

Minimum inhibitory concentrations of the crude extract from *Streptomyces* sp. Go-475 was determined for *C. albicans* ATCC 62376 using the protocol described in NCCLS (2002) and for *B. subtilis* ATCC6633 as described in CLSI (2012). The experiment was performed in Muller Hinton Broth (MHB) for *B. subtilis* whereas; Roswell Park Memorial Institute (RPMI) was used for *C. albicans*. Two fold serial dilutions of the extract in dimethyl sulfoxide were used. Inoculum suspensions of the overnight grown *C. albicans* and *B. subtilis* were prepared in sterile normal saline (0.85%) with direct colony suspension method and the optical density was adjusted to 0.5 Mcfarland standards at a wave length of 530 nm. The inoculum for *B. subtilis* was diluted to a final working count of 2 × 10^8^ CFU/mL whereas for *C. albicans* 2.5 × 10^3^ CFU/mL was used. The experiments were conducted with concentrations of the extract ranging from 0.39 to 800 μg/mL. The cultures were incubated at 37°C for 24 h and inspected visually; the lowest concentrations of the extract that completely inhibited growth of the test organism were considered as MIC values.

### LC-MS profiles of crude extracts of the isolate Go-475

To identify the antimicrobial compounds in the active extracts, an analytical strategy previously established was employed (Ladurner et al., 2017). The crude extracts obtained from both SSF and LSF were diluted to a concentration of 1 mg/mL and first analyzed by high-performance liquid chromatography (HPLC) on an UltiMate 3000 RSLC-series system (Dionex/Thermo Fisher Scientific, Germering, Germany) coupled in parallel to a Corona ultra RS charged aerosol detector (CAD, Dionex/Thermo Fisher Scientific) and an HCT 3D quadrupole ion trap mass spectrometer (MS) equipped with an orthogonal ESI source (Bruker Daltonics, Bremen, Germany). The CAD detector allows the relative quantification of the non-volatile constitutes and thus the identification of the main compounds, while the diode-array detector (DAD) and the MS provide qualitative information. Separation was carried out on a Synergi 4u MAX-RP 80Å, 150 × 4.60 mm, 4 μm HPLC column (Phenomenex) using water and acetonitrile as mobile phase A and B, respectively. The gradient started from 50 to 95% B in 40 min, followed by a washing (10 min at 95% B) and re-equilibration step (10 min at 50% B). The flow rate was 1.0 ml/min and the column oven temperature was set to 35.0°C. After passing the DAD, the eluate flow was split 4:1 between the CAD and the MS, respectively. The CAD nebulizer temperature was 35°C and the ESI ion source was operated as follows: capillary voltage: +3.5/−3.7 kV, nebulizer: 26 psi (N_2_), dry gas flow: 9 L/min (N_2_), and dry temperature: 340°C. Positive and negative ion mode multistage mass spectra up to MS^3^ were obtained in automated data-dependent acquisition (DDA) mode using helium as collision gas, an isolation window of Δ*m/z* = 4, and a fragmentation amplitude of 1.0 V.

In the next step, high-resolution mass spectra were recorded on a maXis HD ESI-Qq-TOF mass spectrometer (Bruker Daltonics) that was also connected to an UltiMate 3000 RSLC-series system. The separation was performed with the above described HPLC methods. The eluate flow was split ~1:8 and the following ESI ion source settings were applied: capillary voltage: ±4.5 kV, nebulizer: 0.8 bar (N_2_), dry gas flow: 7.0 L/min (N_2_), and dry temperature: 200°C. The sum formulas of the detected ions were determined using Bruker Compass DataAnalysis 4.2 based on the mass accuracy (Δm/z ≤ 10 ppm) and isotopic pattern matching (SmartFormula algorithm). All collected MS data are presented in Supplementary Materials.

### Purification of antibacterial compounds and NMR-based structure elucidation

The crude extract (500 mg) was mixed with Silica gel 60 from MERCK (particle size 0.063–0.200 nm for column chromatography) in a proportion 1:2 and packaged in cartridge. For fractionation the PuriFlash column 15 C18 HQ 35G−35.0 g (22 bar) was used with a gradient of 5–98% Methanol/H_2_O in 50 min and a flow of 15 mL/min using an equipment PuriFlash 420. One-hundred and nine Fractions of 0.5 ml were collected, freeze dried, and concentrated in methanol for antimicrobial bioassays. The identification of the antimicrobial compounds in the active fractions was similar as described above for the crude extracts, but the HPLC separation was as given in detail in Ladurner et al. (2017), whereby the gradient used in this study was: 5–95% B in 45 min followed by a washing (10 min at 95% B) and re-equilibration step (10 min at 5% B).

NMR spectra were recorded on a Bruker Avance 500 NMR spectrometer (UltraShield) using a 5 mm switchable probe (TCI Prodigy Kryo-probe head, 5 mm, tripel resonance-invers-detection probe head) with z axis gradients and automatic tuning and matching accessory (Bruker BioSpin). The resonance frequency for ^1^H NMR was 500.13 MHz and for ^13^C NMR 125.75 MHz. All measurements were performed for a solution in fully deuterated chloroform or methanol at 298 K. Standard 1D and gradient-enhanced (ge) 2D experiments, like double quantum filtered (DQF) COSY, HSQC, and HMBC, were used as supplied by the manufacturer. Chemical shifts are referenced internally to the residual, non-deuterated solvent signal for chloroform 1H (δ 7.26 ppm) or methanol (δ 3.31 ppm) and to the carbon signal of the solvent for chloroform 13C (δ 77.00 ppm) or methanol (δ 49.00 ppm). Chemical shifts for compounds 8-O-methyltetrangomycin (**1**) and 8-O-methyltetrangulol (**2**), as well as recorded NMR spectra are given in Supplementary Materials.

### Genome sequencing and analyses

For sequencing of the genome of isolate Go-475, 1 μg of chromosomal DNA was used for generation of a shotgun library (TruSeq PCR-free sequencing library, Illumina Inc.) that was sequenced applying the 2 × 300 bp sequencing protocol on an Illumina MiSeq system as described previously (Zimmermann et al., 2016). The data were assembled using Newbler v.2.8 (Roche), resulting in 423 scaffolds containing 492 contigs. Subsequently, 2 μg of the Go-475 genomic DNA was used for generation of a second shotgun library for sequencing on the MinION system (Oxford Nanopore Technologies). Size-selected DNA-fragments of 6–50 kb were used to create a 1D^2^ sequencing library according to the manufacturer's instructions. Subsequently, the sequencing mix was added to a R9.5 Flowcell for a 24 hours run on the MinION Sequencer. Base calling and data conversion was performed in parallel using Albacore v1.2.4 (Oxford Nanopore Technologies). The sequences were exported in fastq format and used for an assembly with canu v1.5 (Koren et al., 2017). After assembly, the resulting 4 contigs were polished with the short Illumina reads using Pilon (Walker et al., 2014). The final assembly was done manually using Consed (Gordon and Green, 2013) to combine the contigs of the Newbler and canu assemblies. Gene prediction and annotation of the finished genome were performed using the program Prokka (Seemann, 2014). The complete genome sequence was deposited to DDBJ/ENA/GenBank under the accessions no. CP026121.

Analysis of the genome for secondary metabolite BGCs was performed using online version of antiSMASH 4.02 (Blin et al., 2017), followed by manual curation.

## Results

### Actinomycete isolation and characterization

A total of 416 soil actinomycetes were isolated from 13 different understudied habitats in five geographical regions in Ethiopia [see Figure S1 and Table S1 (Supplementary Materials) for locations]. The number of actinomycete cultures obtained varied in each sampling site. Among all the sampling sites, soil samples from Menagesha Suba protected forest (one of the Ethiopian national parks) located in the central region, contributed the highest number of actinomycete cultures (31%), while Ataye located in Ethiopian rift valley area yielded the least number of isolates (7%). Isolates obtained from the Mengaesha Suba protected forest were also rather diverse with respect to colony color, shape, and texture (data not shown).

During the primary antimicrobial activity screening of methanol extracts obtained from solid-state fermentation (SSF) all the 416 isolates were tested for antifungal activity against *C. albicans* and *C. neoformans*. The results showed that the extracts from 101 (24%) isolates were inhibiting the growth of *C. albicans*, extracts from 88 (21%) isolates were inhibiting both *C. albicans* and *C. neoformans*. Extracts from six isolates that were most active in this primary screen, namely those designated Ac-006, Ac-123, Ac-146, Ed-065, Go-475, and Ru-355, were selected for further characterization. Isolates Ac-006, Ac-123, and Ac-146 were obtained from Menagesha suba forest, Ed-065 was obtained from Dengego, Go-475 from Gode and Ru-355 from the Ataye region (see map on Figure S1).

**Figure 1:**
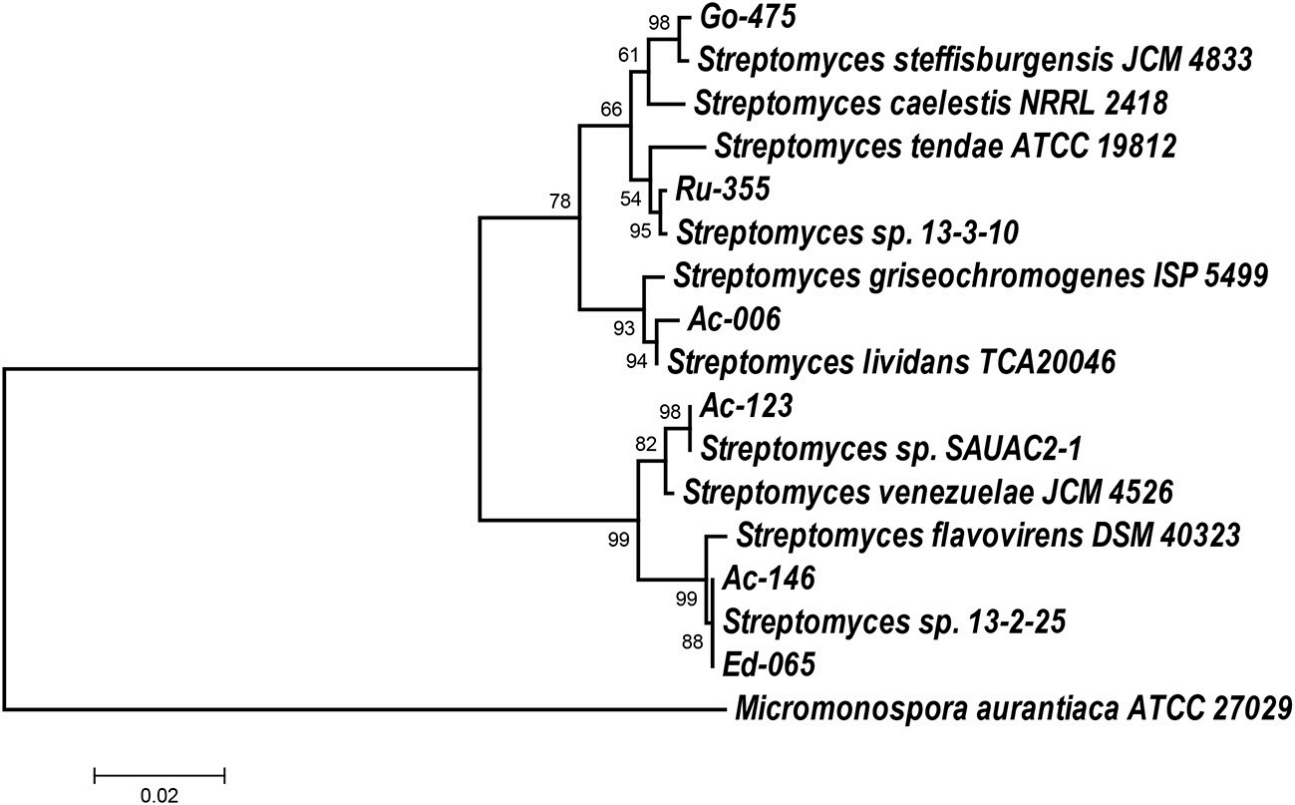
Molecular Phylogenetic analysis by Maximum Likelihood method. The evolutionary history was inferred by using the Maximum Likelihood method based on the Tamura-Nei model (Tamura and Nei, 1993). The tree is drawn to scale, with branch lengths measured in the number of substitutions per site. Evolutionary analyses were conducted in MEGA7 (Kumar et al., 2016). 16S rDNA sequence of *Micromonospora aurantiaca* ATCC 27029 was used as an outgroup.

First, cultural properties of these isolates were determined using standard culture media and guidelines recommended by the International Streptomyces Project. The data obtained in these experiments are presented in Tables S2, S3 (Supplementary Materials). Selected isolates were identified on the genus level using 16S rDNA gene sequences obtained after amplification from genomic DNA. All sequences were analyzed using the RDP database, which clearly indicated that all analyzed isolates belong to the genus *Streptomyces*. Next, the phylogenetic relationships of the isolates to type strains and best matches according to the nucleotide BLAST search were inferred using the Maximum Likelihood algorithm in MEGA 7 software (Kumar et al., 2016). According to the phylogenetic tree presented in Figure 1, isolates Ac-006, Ac-123, Go-475, and Ru-355 all belonged to distinct clades, while Ed-065 and Ac-146 showed identical 16S rRNA gene sequences and thus appeared to belong to the same clade.

Growth characteristics of the six selected *Streptomyces* spp. were examined at different temperatures and pH levels. The results showed that most cultures were able to grow well between 20 and 35°C and at pH values between 6.5 and 8.5. The observed optimum growth for most of the isolates was at 30°C and pH 7.5. Most isolates were positive for starch hydrolysis, chitinase, oxidase, and catalase tests, and showed a negative result for indole production. However, Ac-006 was oxidase negative (Table S4, Supplementary Materials). *Streptomyces* pp. Ac-029, Ac-123, Ac-146, and Go-475 were found to be positive for melanin production.

### Bioactivities of six selected *Streptomyces* isolates

The methanol extract from isolate Go-475 manifested the highest antifungal activity, followed by those of Ru-355 and Ed-065 (Table 1). The bioactivity spectrum of the crude extracts evaluated against bacterial pathogens generally showed better activities against Gram-positive bacteria, with the highest activity detected for the extract from Go-475 against *B. subtilis*, followed by those from Ru-355 and Ed-065. Only few isolates showed activity against Gram-negative bacteria in these tests; most pronounced was that of Ru-355 (Table 1). Interestingly, isolates Ac-146 and Ed-065 which, according to the 16S rDNA sequences, are closely related, displayed different bioactivity profiles. As one of the most bioactive isolates, strain Go-475 was subjected to the liquid-state fermentation (LSF) and the obtained extract also screened for antimicrobial activities. Interestingly, in contrast to the extract obtained after SSF no activity against yeasts could be detected for the extract obtained after LSF, while a significant activity against Gram-positive *B. subtilis* was observed for both extracts (data not shown).

**Table 1:** Activity profiles of the crude extracts from selected isolates against *Candida albicans* and *Cryptococcus neoformans*, as well as two Gram-positive and three Gram-negative bacterial pathogens.

**Isolate**	**Pathogenic yeasts**		**Gram-positive bacteria**		**Gram-negative bacteria**		
	***C. albicans***	***C. neoformans***	***S. aureus***	***B. subtilis***	***E. coli***	***S. typhimurium***	***S. boydi***
Ac-006	6 ± 1	5 ± 1	–	6 ± 2	5 ± 1	5 ± 0	–
Ed-065	13 ± 0	11 ± 1	12 ± 1	11 ± 2	–	–	–
Ac-123	8 ± 1	6 ± 2	–	–	–	–	–
Ac-146	7 ± 1	9 ± 2	–	–	–	–	–
Ru-355	27 ± 2	28 ± 1	14 ± 1	19 ± 1	18 ± 1	18 ± 1	16 ± 1
Go-475	31 ± 2	27 ± 1	21 ± 2	21 ± 1	–	–	11 ± 1

*Numbers indicate diameters of inhibition zones (mm)*.

Minimal inhibitory concentration for the Go-475 extract prepared after SSF was determined for *C. albicans* and *B. subtilis* (see Materials and Methods). The result revealed that MIC for *C. albicans* at 100 and 50 μg/mL for *B. subtilis*.

### Identification and structure elucidation of the anti-bacterial compounds produced by *Streptomyces* sp. Go-475

The methanolic LSF extract of strain Go-475 showing pronounced activity against *B. subtilis* was fractionated using Flash chromatography. Fractions 24 (27.41–28.53 min) and 42 (49.20–50.32 min) were found to be responsible of the bioactivity against *B. subtilis*. These fractions were analyzed using HPLC-CAD/MS and high resolution LC-MS as described in Ladurner et al. (2017). Fraction 42 contained several similarly abundant compounds, which were tentatively identified as (branched) fatty acids and not further analyzed. In fraction 24, on the other hand, only one major constituent was detected.

High-resolution ESI-Qq-TOF-MS spectra, obtained by direct infusion of fraction 24, showed the main compound as [M+H]^+^ ion at *m/z* 337.1073 (calculated for C_20_H_17_${\text{O}}_{5}^{+}$,*m/z* 337.1071, Δ = 0.8 ppm) and as [M+Na]^+^ ion at *m/z* 359.0891 (calculated for C_20_H_16_O_5_Na^+^,*m/z* 359.0890, Δ = 0.2 ppm). The HRMS/MS-spectra of the [M+H]^+^ ion were not very informative, which is typical for very stable aromatic systems like angucycline antibiotics. However, the abundant loss of H_2_O indicated an aliphatic hydroxyl-group (see Figures S2, S3). All these data fit nicely to the known compound 8-O-methyltetrangomycin (C_20_H_16_O_5_), but due to the lack of a reference standard or published MS/MS spectra, this preliminary identification could not be confirmed without further structure elucidation by 1D and 2D NMR spectroscopy (see Materials and Methods for details).

**Figure 2:**
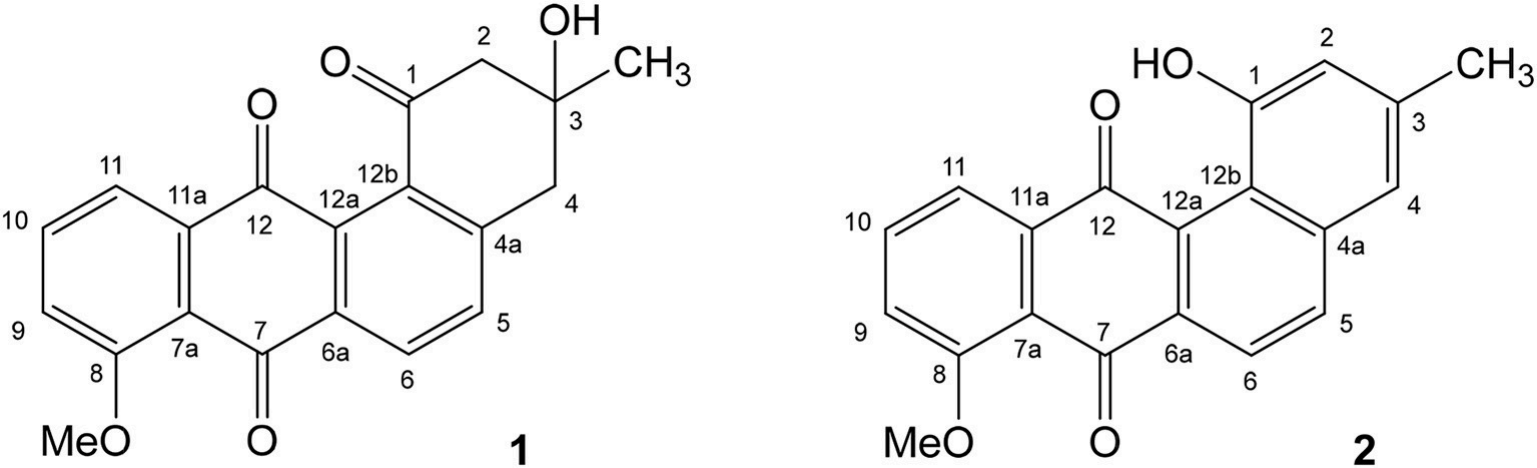
Structure of the antibacterial compounds 8-O-methyltetrangomycin **(1)** and 8-O-methyltetrangulol **(2)** produced by *Streptomyces* sp. Go-475.

When the NMR analysis was executed in deuteromethanol, we obtained 8-O-methyltetrangomycin (**1**) as the result, while measuring the sample in deuterochloroform indicated a complete conversion to 8-O-methyltetrangulol (**2**) (Figure 2). Obviously, the conversion of **1**–**2** was catalyzed by traces of hydrochloric acid present in deuterochloroform. Both compounds showed typical signals of a methoxyanthraquinone system. The different oxidation state of the six-membered ring D was indicated in 8-O-methyltetrangomycin by the two methylene carbons C-2 and C-4, while in 8-O-methyltetrangulol two olefinc methine signals were detected for C-2 and C-4. Assignment of the signals was confirmed by COSY, HSQC, and HMBC measurements. Comparison of the spectral data of **1** and **2** with literature values confirmed the structure elucidation (Kesenheimer and Groth, 2006; Ding et al., 2009).

### *Streptomyces* sp. Go-475 genome analyses

The genome of *Streptomyces* sp. Go-475 was completely sequenced (see Materials and Methods for details), revealing a linear chromosome of 8,570,609 bp, featuring a G+C content of 71.96%, 7,571 predicted coding sequences, 83 t(m)RNA genes, and six *rrn* operons. In addition, a total of 19 RNA features (5′-UTRs, riboswitches, ncRNAs, etc) were identified based on hits against RFAM. The inverted terminal repeats of the chromosome were found to be rather short, with a length of just 6,902 bp, which is unusual for *Streptomyces*.

The genome sequence of *Streptomyces* sp. Go-475 shows significant sequence similarity to that of *Streptomyces olindensis* DAUFPE 5622 (GenBank accession number JJOH00000000), which is ca 0.8. Mb larger. Apparently, the core genomes of these two strains are very similar, with the ANI (Average Nucleotide Identity) of 94.56% over a length of 6,649,500 bp. Synteny plot of the two genomes (Figure S1, Supporting Information) clearly demonstrates close relatedness of *Streptomyces* sp. Go-475 and *S. olindensis*, despite the genome of the latter being rather fragmented (draft quality sequence). This fact raises the issues of biogeography and distribution of microorganisms, since these strains were isolated on two different continents separated by a vast expanse of water.

**Figure 3:**
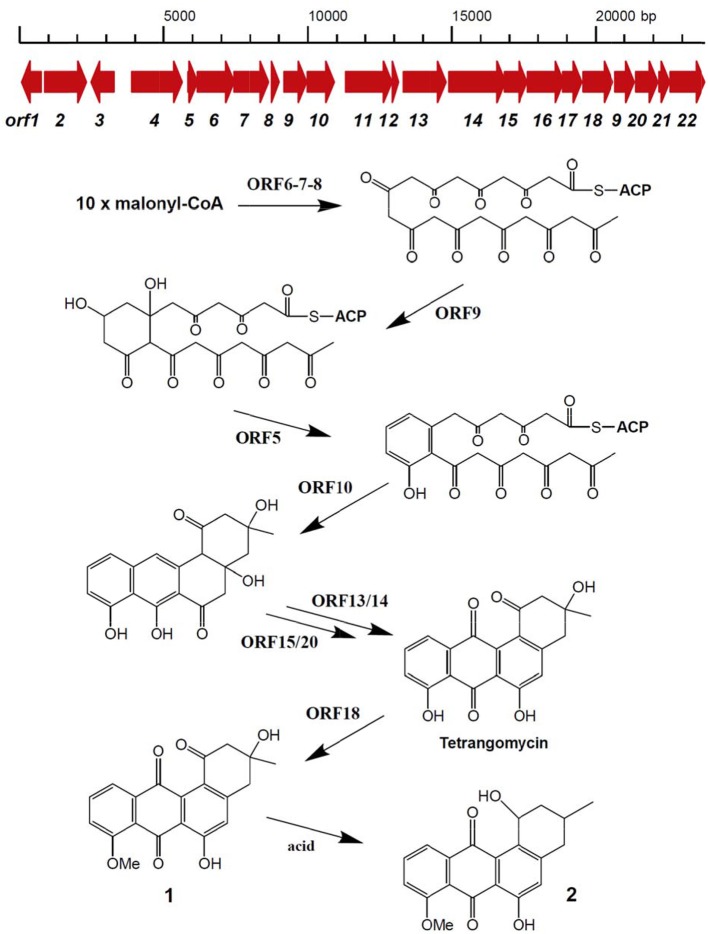
Organization of *Streptomyces* sp. Go-475 cluster 31 presumably involved in angucycline biosynthesis, and suggested biosynthetic pathway up to 8-O-methyltratrangomycin.

Secondary metabolite BGCs were identified in the genome of *Streptomyces* sp. Go-475 using antiSMASH 4.02 followed by manual curation to split apparently disparate BGCs suggested by the software as being single ones. In total, 36 BGCs were identified (Table 2), 27 of which were also present in the genome of *S. olindensis* DAUFPE 5622 (Rojas et al., 2014). The latter streptomycete was isolated in 1960s from a soil sample collected in Brazil, and was shown to produce cytotoxic glycosylated anthracyclines named cosmomycins. The BGC for cosmomycin could be readily identified in the genome of Go-475 (cluster 23, Table 3). Cluster 9, of the ectoine type, most likely is responsible for the biosynthesis of ectoine and, possibly, hydroxyectoine and betaine (see next section). Beside this, BGCs for geosmin (cluster 28) and the angucyclines (cluster 31, Figure 3) were also identified. The latter cluster is apparently absent in the *S. olindensis* genome, and contains genes encoding polyketide synthases type II (PKSII) along with some modification enzymes. Interestingly, although the BGC for both angucyclines were previously identified (Hong et al., 1997), and core genes identified for this type of compounds (Palmu et al., 2007) most of their products showed <80% homology to the corresponding enzymes encoded by cluster 31. The latter cluster also contains some unique genes not found in other angucycline BGCs (Table 4), suggesting that 8-O-methyltetrangomycin and 8-O-methyltetrangulol (see below) could be mere precursors for more complex novel compounds. Based on the gene annotation for cluster 31 and current knowledge on the biosynthesis of angucyclines, a biosynthetic pathway leading to 8-O-methyltetrangomycin could be suggested (Figure 3). The building blocks needed for the biosynthesis, malonyl-CoAs, are likely to be afforded via carboxylation of acetyl-CoA by ORF4, ORF11, and ORF12. The minimal polyketide synthase II (ORFs 6,7, and 8) performs a decarboxylative condensation of the acetate units into a poyketide chain, which undergoes reduction by ORF9 and cyclization/dehydration by ORF5 resulting in the formation of the first aromatic ring. Subsequent cyclizations by ORF10 lead to the formation of an angucycline core. Further modification steps are likely to be accomplished by oxygenases ORF13/ORF14 and oxidoreductases ORF15/ORF20 to afford tetrangomycin. ORF18 performs O-methylation of tetrangomycin at position C8 using S-adenosylmethionine, synthesized by ORF22, as a methyl group donor to generate compound **1**. The latter is spontaneously converted to **2** in the presence of an acid.

**Table 2:** Secondary metabolite biosynthesis gene clusters identified in the genome of *Streptomyces* sp. Go-475 with antiSMASH 4.02 (manually curated).

**No**	**Cluster type**	**Presence in another bacterium**	**Putative product**
1	PKSI-NRPS	*Streptomyces olindensis* DAUFPE 5622	Glycosylated PK-NRS peptide hybrid
2	Melanin	Multiple *Streptomyces* spp.	Melanin
3	Nrps	*Streptomyces olindensis* DAUFPE 5622	NRS peptide
4	Unknown	*Streptomyces olindensis* DAUFPE 5622	Amino acid-based product
5	Terpene	*Streptomyces olindensis* DAUFPE 5622	Putative sesquiterpene
6	Lantipeptide	*Streptomyces viridochromogenes* DSM 40736	SapB/AmfS family lantipeptide
7	PKSIII	*Streptomyces* sp. XY006	1,3,6,8-tetrahydroxynaphthalene
8	Lassopeptide	*Streptomyces olindensis* DAUFPE 5622	Lassopeptide
9	Ectoine	*Streptomyces olindensis* DAUFPE 5622	**Ectoine, betaine**
10	Terpene	*Streptomyces olindensis* DAUFPE 5622	Putative isoprenoid
11	PKSI-NRPS	*Streptomyces chartreusis* NRRL 12338	PK-NRS peptide hybrid
12	NRPS	*Streptomyces olindensis* DAUFPE 5622	Glycosylated NRS peptide
13	Terpene	*Streptomyces olindensis* DAUFPE 5622	Isoprenoid
14	Melanin	*Streptomyces* sp. S10(2016)	Melanin
15	Lassopeptide	*Streptomyces chartreusis* NRRL 12338	Lassopeptide
16	Siderophore	Multiple *Streptomyces* spp.	**Desferrioxamine B**
17	Butyrolactone	*Streptomyces olindensis* DAUFPE 5622	Butyrolactone
18	PKSI-NRPS	*Streptomyces olindensis* DAUFPE 5622	PK-NRS peptide hybrid
19	Phosphonate	*Streptomyces olindensis* DAUFPE 5622	Phosphonate metabolite
20	Terpene	–	Terpenoid
21	Aminocyclitol	*Streptomyces olindensis* DAUFPE 5622	Aminocyclitol
22	NRPS	*Streptomyces vitaminophilus* DSM 41686	NRS peptide
23	PKSII	*Streptomyces olindensis* DAUFPE 5622	**Cosmomycins**
24	Terpene	*Streptomyces olindensis* DAUFPE 5622	**Albaflavenone**
25	Terpene	–	Terpenoid (polyprenyl)
26	Siderophore	*Streptomyces olindensis* DAUFPE 5622	Siderophore
27	Bacteriocin	*Streptomyces olindensis* DAUFPE 5622	Bacteriocin
28	Terpene	Multiple *Streptomyces* spp.	**Geosmin**
29	PKSI-NRPS	*Streptomyces africanus* DSM 41829	PK-NRS peptide hybrid
30	Siderophore	*Streptomyces olindensis* DAUFPE 5622	Siderophore
31	PKSII	–	**Angucyclines**
32	Terpene	*Streptomyces olindensis* DAUFPE 5622	Hopanoids
33	PKSI	*Streptomyces olindensis* DAUFPE 5622	PK
34	Bacteriocin	Multiple *Streptomyces* spp.	Bacteriocin
35	Lantipeptide	Multiple *Streptomyces* spp.	SapB/AmfS family lantipeptide
36	PKSI	*Streptomyces olindensis* DAUFPE 5622	PK

*NRS, non-ribosomally synthesized; PK, polyketide. Shaded cells show gene clusters not present in the genome of S. olindensis DAUPFE 5622. Products in bold text are inferred with certainty based on >90% identity of the gene products to those of the verified gene clusters or experimental data (e.g., angucyclines)*.

**Table 3:** Compounds tentatively identified by high-resolution LC-MS in the crude extracts of *Streptomyces* sp. Go-475 after liquid (LSF) and solid (SSF) media fermentations.

**No**	**Accurate mass, Da**	**Compound ID**	**Type of fermentation**	
			**LSF**	**SSF**
1	142.0745	Ectoine	+	+
2	158.0694	Hydroxyectoine	+	+
3	1180.5236	Anthracycline antibiotic	+	+
4	1184.5536	Anthracycline antibiotic	+	+
5	1188.5830	Cosmomycin D	+	+
6	117.0794	Betaine	+	
7	898.4480	Anthracycline antibiotic	+	
8	912.4634	Anthracycline antibiotic	+	
9	928.4586	Anthracycline antibiotic	+	
10	944.4530	Anthracycline antibiotic	+	
11	960.4478	Anthracycline antibiotic	+	
12	755.3508	Cosmomycin A	+	
13	771.3462	Cosmomycin B	+	
14	787.3409	Anthracycline antibiotic	+	
15	801.3566	Anthracycline antibiotic	+	
16	336.1008	8-O-Methyltetrangomycin	+	
17	164.0474	4-Methoxy-1(3H)-isobenzofuranone^*^		+
18	150.0682	3-Phenylpropionic acid^*^		+
19	318.0897	8-O-Methyltetrangulol	+	+
20	218.1675	Albaflavenone	+	+
21	292.1674	Dehydrocineromycin B^*^		+

*Data presented in the order of the retention time in LC. Compounds in bold font have been confirmed by NMR-assisted structure elucidation*.

* *Not supported by genome analysis*.

**Table 4:** Characteristics of the gene cluster 31 of *Streptomyces* sp. Go-475 presumably involved in the biosynthesis of 8-O-Methyltetrangomycin.

**Gene**	**Putative product**	**Putative function**
*orf1*	TetR family transcriptional regulator	Efflux regulation
*orf2*	MFS family transporter	Compounds' efflux
*orf3*	Response regulator, LuxR family	Regulation of biosynthesis
*orf4*	Acetyl-CoA carboxylase	Biosynthesis of malonyl-CoA
*orf5*	Polyketide cyclase	Cyclization of the polyketide chain
*orf6*	Beta-ketoacyl synthase	Polyketide biosynthesis
*orf7*	Beta-ketoacyl synthase CLF	Polyketide chain length factor
*orf8*	Acyl carrier protein	Polyketide biosynthesis
*orf9*	3-oxoacyl-ACP reductase	1st reductive step in angucycline biosynthesis
*orf10*	Polyketide cyclase	1st cyclization/dehydration
*orf11*	acyl-CoA carboxylase subunit beta	Biosynthesis of malonyl-CoA
*orf12*	acyl-CoA carboxylase subunit epsilon	Biosynthesis of malonyl-CoA
*orf13*	Monooxygenase FAD-binding	Oxygenation at C12?
*orf14*	Monooxygenase FAD-binding	Oxygenation at C12?
*orf15*	SDR oxidoreductase	unknown
*orf16*	MFS family transporter	Compounds' efflux
*orf17*	Antibiotic biosynthesis monooxygenase	unknown
*orf18*	O-methyltransferase	Conversion of Tetrangomycin to 8-O-methyltatrangomycin
*orf19*	Antibiotic biosynthesis monooxygenase	unknown
*orf20*	SDR oxidoreductase	unknown
*orf21*	Antibiotic biosynthesis monooxygenase	unknown
*orf22*	S-adenosylmethionine synthetase	Provision of a methyl donor for O-methyltransferase (ORF18)

The genome of isolate Go-475 harbors several BGCs, homologs of which could not be identified in other genomes available in the public databases. These include the above mentioned angucycline BGC, as well as clusters 20 and 25 containing terpene synthase genes. Terpene synthase encoded by cluster 20 is highly unusual in that it contains a N-terminal carboxypeptidase regulatory-like domain, while its terpene cyclase domain shares only 76% homology with the best database hit. This BGC also encodes a putative trans-isoprenyl diphosphate synthase, which may provide precursor for the cognate terpene cyclase.

Cluster 25 contains genes encoding polyprenyl synthase (only 67% identity to the closest match in the database), IspH homolog (last enzyme in isopentenyl pyrophosphate biosynthesis), methyltransferase, prenyltransferase, and MFS superfamily transporter. It is not clear at the moment, what kind of terpenoid is specified by this BGC.

### Analytical LC-MS profiles of methanol extracts of the isolate Go-475

Since the extracts of *Streptomyces* sp. Go-475 displayed high antimicrobial activities, which differed depending on the mode of fermentation (solid or liquid media), we decided to investigate both extracts using high-resolution LC-MS (see Experimental Procedures for details). The data were analyzed in two different ways, untargeted and targeted. First, the mass spectra of all main compounds were interpreted and potential sum formulae predicted. Both the accurate masses and predicted chemical formulae were then queried against the Dictionary of Natural Products (DNP, contains data on ca. 250.000 natural products). Secondly, metabolites that were predicted based on homology of the sequenced BGCs to known ones, namely cahuitamycins A-C, desferrioxamine B, cosmomycin D, flaviolin, albaflavenone, ectoine, and betaine, were searched for by creating extracted ion chromatograms of the corresponding [M+H]^+^ (for all of them), [M+2H]^2+^ (for cosmomycin D), and [M-H]^−^ (for flaviolin and ectoine) ions. The results of this analysis are presented in Table 3 and Table S5.

As expected from the identified PKSII (clusters 23 and 31), numerous anthracyclines could be identified, which are most likely responsible for the activity against Gram-positive bacteria. Besides compounds matching the sum formulae of derivatives of tetrangulol (Kuntsmann and Mitscher, 1966) and of cosmomycin A, B, and C, several other potential anthracyclines, whose assignment was ambiguous or which could not be matched to known compounds in the DNP, were detected. Production of the known secondary metabolite 8-O-methyltetrangomycin (Shigihara et al., 1988) could only be detected in the LSF extract. However, previous studies showed non-enzymatic conversion of 8-O-methyltetrangomycin (**1**) to 8-O-methyltetrangulol (**2**) (Figure 2) under acidic conditions, so that this finding does not necessarily reflect differences in the biosynthesis (Grabley et al., 1991).

Furthermore, the known osmolytes ectoine, hydroxyectoine, and betaine, which serve as osmotic stress protectants, were tentatively identified by MS analyses. All the above mentioned compounds except 8-O-methyltetrangulol were much more abundant or exclusively found in the LSF extract. However, this might be due to different extraction efficiencies for these very polar compounds between the liquid and solid medium and does not necessarily reflect altered biosynthesis.

From the remaining predicted compounds, only albaflavenone could be tentatively identified with low abundance. On the other hand, three of the main compounds produced during SSF of strain Go-475 matched to known secondary metabolites reported from *Streptomyces* spp., namely 4-methoxy-1(3H)-isobenzofuranone, 3-phenylpropionic acid, and dehydrocineromycin B. However, no BGCs in the genome could be associated with these three compounds. While an unusual mode of biosynthesis of these metabolites, which made it impossible to unequivocally propose their BGCs, cannot be excluded, it is much more likely that they represent false positive hits at the DNP, which would not be surprising considering their rather common sum formulae. Most likely, they represent previously undetected cosmomycin analogs or precursors.

In addition to the tentatively identified compounds listed in Table 3 and obvious primary metabolites, such as fatty acids and their derivatives, extracts from both LSF and SSF contained a number of substances that could not be identified using DNP query (data not shown). Notably, there were many more such compounds in the SSF extract compared to those in the LSF extract. As suggested by the high number of BGCs that cannot be linked to known molecules, some of these unidentified substances might be novel and warrant further studies with regard to their origins and bioactivities. In particular, none of the tentatively identified secondary metabolites could be clearly associated with the strong antifungal activity shown for the SSF extract, suggesting that a novel compound might be responsible for this bioactivity.

## Discussion

The threat of resistance of infectious agents to currently available antibiotics has become a global challenge, particularly in the developing world (Demain, 2009; Berdy, 2012; Hamers et al., 2012). The current study was focused on the isolation and characterization of actinomycetes from previously unstudied habitats in Ethiopia in hope to identify new antimicrobial agents. Several bioactive isolates originating from different locations were taxonomically identified and their extended antimicrobial spectra investigated. Characterization of bioactive isolates revealed them being dominated by the representatives of the genus *Streptomyces*. Likewise, previous studies reported that members of the genus *Streptomyces* isolated from the terrestrial or aquatic environments are responsible for the highest share (in terms of novelty and frequency) of antimicrobial substance production (Basilio et al., 2003; Berdy, 2005; Bruntner et al., 2005; Demain, 2009; Rao et al., 2012). This pronounced antimicrobial production capacity in the genus *Streptomyces* might be related to the presence of multiple secondary metabolite BGCs in relatively large genomes, ranging in size from ca 6 to 11 Mb (Nett et al., 2009).

From the six selected Ethiopian isolates, the best ones in terms of activity spectra were *Streptomyces* spp. Go-475 and Ru-355 isolated from Gode (in Somali region) and Ataye (Rift valley area, in Amhara region), respectively. The extract from isolate Go-475 showed considerable activities against Gram-positive bacteria such as *S. aureus* and *B. subtilis*, while Ru-355 was also active against the Gram-negative bacteria *E. coli* and *S. typhimurium*. It is also worth noting that two isolates, Ac-146 and Ed-065, displaying 100% identical 16S rRNA gene sequences, have different bioactivity profiles (Table 2). This suggests that the environment where the strains were isolated influenced their secondary metabolite biosynthesis potential, most likely via acquisition of specific biosynthetic gene clusters. A similar trend was demonstrated for the marine actinomycete *Salinispora*, where extensive horizontal gene transfer was shown to account for the majority of secondary metabolite biosynthetic pathways (Ziemert et al., 2014).

In this work, we have focused on the identification of antibiotics produced by the isolate Go-475. First, the compounds active against *B. subtilis* were purified from the liquid culture of this isolate, and their chemical structures determined by NMR. Both compounds, identified as 8-O-methyltetrangomycin (**1**) and 8-O-methyltetrangulol (**2**) have been described earlier (Shigihara et al., 1988). Published bioactivity data for these compounds (MIC> 100 μg/mL and > 50 μg/mL for 1 and 2, respectively, against *C. albicans*; 100 μg/mL and 12.5 μg/mL, respectively, for the same compounds against *B. subtilis*) and our own MIC data for crude extract suggest, that other compounds in the extract may also contribute to its bioactivity. To gain a deeper insight into the secondary metabolite profiles of Go-475, this isolate was grown on solid (SSF) and in liquid media (LSF), and the extracts from these cultures were compared using LC-MS analysis. As expected, the metabolite profiles of the extracts were quite different, and, using high-resolution MS, we were able both, to identify known and detect potentially unknown metabolites. Notably, considerably more presumably novel secondary metabolites were found in the SSF extract suggesting that this method of cultivation shall be further exploited in the future bioprospecting efforts. The most probable explanation for this observation is that streptomycetes in soils usually dwell on solid particles also occupied by other microorganisms, with whom they compete for nutritional sources. It thus appears likely, that they tend to produce a wider array of bioactive secondary metabolites in SSF, where conditions more closely resemble native ones compared to artificial LSF.

Genome sequencing and analysis of the isolate Go-475 revealed a linear chromosome, typical for streptomycetes. However, it is noteworthy, that terminal inverted repeats of the Go-475 chromosome are unusually short, ca 7 kb, while in other streptomycetes they range from 30 kb to 1 Mb (Chen et al., 2002; unpublished data). At least 36 BGCs could be identified in the genome, some of which appear to be unique, and may encode novel secondary metabolites. Even seemingly known gene clusters, e.g., that for angucyclines biosynthesis, contain unique additional genes, which may be implicated in modifications never before seen for this kind of molecules. Interestingly, the majority of the BGCs are present in a streptomycete isolated from Brazilian soil, raising interesting questions regarding distribution of a common ancestor of these bacteria.

## Conclusion

This study demonstrates for the first time the potential of streptomycetes isolated from unique environmental niches in Ethiopia to produce antimicrobial compounds. This work also sets a stage for a comprehensive investigation of *Streptomyces* sp. Go-475, as well as other Ethiopian isolates, in terms of identification and characterization of new bioactive secondary metabolites and their biosynthetic gene clusters. In particular, unique biosynthetic gene clusters, which have not been previously detected in bacteria, may become targets for genome mining via heterologous expression or activation aimed at discovery of new molecules with therapeutic potential. Moreover, high degree of similarity between the genomes of Go-475 isolate and a streptomycete isolated in Brazil raises interesting questions about biogeographical distribution of these bacteria. Noteworthy is the number of identical secondary metabolite BGCs present in these bacteria, suggesting that these play an important role in their life styles irrespective of the environments.

## Author contributions

MK, DA, MZ, JR, CR, and SBZ conceived the project, conducted data analysis, and wrote the manuscript. MK and JG-G performed most of the experiments on sample collection, fermentations, bioassays, microscopy, and taxonomic identification. MZ, V-KW, and JR acquired and analyzed LC-MS data. EU acquired and analyzed NMR data. CR, TB, and JK sequenced, assembled, and analyzed the genome. All authors reviewed and discussed the manuscript, and contributed to its writing.

### Conflict of interest statement

The authors declare that the research was conducted in the absence of any commercial or financial relationships that could be construed as a potential conflict of interest.
