# Supplementary material for: Streptomyces spp. From Ethiopia Producing Antimicrobial Compounds: Characterization via Bioassays, Genome Analyses, and Mass Spectrometry
Source: Front Microbiol. 2018 Jun 12;9:1270. doi: 10.3389/fmicb.2018.01270 (PMC6007079; doi:10.3389/fmicb.2018.01270)
Supplement: Supplementary file 1 [file Data_Sheet_1.docx]

**Supplementary Materials**

***Streptomyces* spp. from unique environmental niches in Ethiopia: isolation and evaluation of biotechnological potential via bioassays, genome analyses, and mass spectrometry**

Moges Kibret^1^, Jaime Felipe Guerrero-Garzón^2^, Ernst Urban^3^, Martin Zehl^4^, Valerie-Katharina Wronski^2^, Christian Rückert*^5^*, Tobias Busche^*,^*^5^*, Jörn Kalinowski*^5^*, Judith M. Rollinger^2^, Dawit Abate^1^, Sergey B. Zotchev^2^*

*^1^Microbial, Cellular and Molecular Biology Department, College of Natural Science, Addis Ababa University, P.O. Box 1176, Addis Ababa, Ethiopia.*

*^2^Department of Pharmacognosy, University of Vienna, 1090 Vienna, Austria*

*^3^Department of Pharmaceutical Chemistry, University of Vienna, 1090 Vienna, Austria*

*^4^Department of Analytical Chemistry, Faculty of Chemistry, University of Vienna, 1090 Vienna, Austria.*

*^5^Center for Biotechnology, Bielefeld University, Universitätsstraße 27, 33615 Bielefeld, Germany*

**Present address: Institute for Biology-Microbiology, Freie Universität Berlin, Berlin, Germany*

**Table S1.** Sampling sites in different geographical regions in Ethiopia.

| SN | Geographical Regions | Specific soil sampling points | Longitude | Latitude |
| --- | --- | --- | --- | --- |
| 1 | Central to south west | Menagesha Suba | 38° 50ˈ54ˈˈ | 8° 56ˈ12ˈˈ |
|  |  | Gera | 37° 7ˈ23ˈˈ | 7° 25ˈ12ˈˈ |
|  |  | Qotie | 38° 38ˈ20ˈˈ | 6° 24ˈ32ˈˈ |
| 2 | Rift valley area | Ataye | 40° 13ˈ12ˈˈ | 10° 14ˈ11ˈˈ |
| 3 | Northern | Guassa | 40° 3ˈ56ˈˈ | 10° 17ˈ40ˈˈ |
|  |  | Mollale | 39° 56ˈ56ˈˈ | 10° 2ˈ45ˈˈ |
|  |  | Tara Gedam | 38° 1ˈ48ˈˈ | 12° 3ˈ21ˈˈ |
|  |  | Zegae Gedam | 37° 35ˈ3ˈˈ | 11° 50ˈ36ˈˈ |
| 4 | Southern Region | Wondogenet forestry | 38° 55ˈ33ˈˈ | 7° 5ˈ54ˈˈ |
|  |  | Wondogenet | 38° 12ˈ48ˈˈ | 7°4ˈ45ˈˈ |
|  |  | Negelle Borena | 39° 57ˈ27ˈˈ | 5°20ˈ9ˈˈ |
| 5 | Eastern Region | Gode | 43° 52ˈ23ˈˈ | 5° 55ˈ41ˈˈ |
|  |  | Dengego | 42° 2ˈ55ˈˈ | 9° 34ˈ27ˈˈ |

**Table S2.** Cultural properties and spore diameter of the isolates on starch casein agar media and their source environment

| **Isolates** | **Morphological and cultural characteristics of the isolates on starch Casein Agar medium** | | | | | |
| --- | --- | --- | --- | --- | --- | --- |
|  | Geographical regions | Specific Sites | Aerial  mycelium | Substrate  mycelium | Spore chain morphology | Mean spore diameter in µm |
| Ac-006 | Central Ethiopia | Menagesha | RAL1013 Oyster white | RAL1002 Sand yellow | Rectiflexibiles | 2.61±.27de |
| Ac-029 | Central Ethiopia | Menagesha | RAL9003 Signal white | RAL5008 Grey blue | Rectiflexibiles | 3.01±.39cd |
| Ed-065 | Eastern | Dengego | RAL1013 Oyster white | RAL1034 Pastel yellow | Spiral | 3.85±.57b |
| Ac-123 | Central Ethiopia | Menagesha | RAL9001 Cream | RAL7042 Traffic grey B | Retinaculiaperti | 5.92±.02a |
| Ac-125 | Central Ethiopia | Menagesha | RAL9016 Traffic white | RAL2010 Signal orange | Rectiflexibiles | 1.96±.04e |
| Ac-146 | Central Ethiopia | Menagesha | RAL9016 Traffic white | RAL6006 Grey olive | Retinaculiaperti | 5.88±.01a |
| Rv-355 | Rift valley | Ataye | RAL 9003 Signal white | RAL7039 Quartz grey | Rectiflexibiles | 2.78±.71d |
| Ac-464 | Central Ethiopia | Menagesha | RAL 4009 Pastel violet | RAL5012 Light blue | Retinaculiaperti | 3.63±.23bc |
| Go-466 | Eastern | Gode | RAL1018 Zink yellow | RAL 9016 Traffic white | Spiral | 2.90±.00cd |
| Go-475 | Eastern | Gode | RAL9003 Signal white | RAL7030 Pebble grey | Spiral | 2.64±.76de |

**Table S3.** Cultural characteristics of isolates observed on various international streptomyces project (ISP) media.

| **Isolates** | **ISP Media** | **Substrate Mycelium** | **Aerial mycelium** | **Degree of Growth** | **Diffusible pigments** |
| --- | --- | --- | --- | --- | --- |
| Ac-006 | ISP 2 | RAL 2009 Traffic Orange | RAL7047 Telegrey4 | Good | _ |
|  | ISP 3 | RAL 8024 Beige brown | RAL 1013 Oyster white | Abundant | _ |
|  | ISP 4 | RAL 1017 Saffron yellow | RAL 7047 Tele grey 4 | Moderate | _ |
|  | ISP 5 | RAL 1015 Light Ivory | RAL 1002 Sand yellow | Moderate | _ |
| Ac-029 | ISP 2 | RAL 5004 Black blue | RAL7001 Silver grey | Good | Pale green |
|  | ISP 3 | RAL 5008 Grey blue | RAL 9002 Grey white | Abundant | Olive green |
|  | ISP 4 | RAL 5011 Steel blue | RAL 7035 Light grey | Good | Olive Brown |
|  | ISP 5 | RAL 5008 Grey blue | RAL 5021 Water blue | poor | _ |
| Ed-065 | ISP 2 | RAL 7013 Brown grey | RAL 9003 Signal white | Moderate | _ |
|  | ISP 3 | RAL 6006 Grey olive | RAL 9003 Signal white | Abundant | Signal Brown |
|  | ISP 4 | RAL 2011 Deep orange | RAL 9001 Cream | Good | _ |
|  | ISP 5 | RAL 2000 Yellow orange | RAL 9001 Cream | Good | _ |
| Ac-123 | ISP 2 | RAL 8004 Cooper brown | RAL7035 Light grey | Moderate | Clay brown |
|  | ISP 3 | RAL 5011 Steel blue | RAL 7047 Tele grey 4 | Abundant | Green brown |
|  | ISP 4 | RAL 8022 Black brown | RAL 7001 Silver grey | Good | Signal Green |
|  | ISP 5 | RAL 1015 Light Ivory | RAL 9001 Cream | Moderate | _ |
| Ac-125 | ISP 2 | RAL 7039 Quartz grey | RAL 9010 Pure white | Good | _ |
|  | ISP 3 | RAL 8019 Grey brown | RAL 1013 Oyster white | Excellent | Chrome Green |
|  | ISP 4 | RAL 2011 Deep orange | RAL 1013 Oyster white | Moderate | _ |
|  | ISP 5 | RAL 1017 Saffron yellow | RAL 1013 Oyster white | Good | _ |
| Ac-146 | ISP 2 | RAL 8000 Green brown | RAL 9016 Traffic white | Moderate | Green Brown |
|  | ISP 3 | RAL 7024 Graphite grey | RAL 9002 Grey white | Moderate | Red Orange |
|  | ISP 4 | RAL 7002 Olive grey | RAL 9003 Signal white | Good | _ |
|  | ISP 5 | RAL 1018 Zink yellow | RAL 9010 Pure white | poor | _ |
| Rv-355 | ISP 2 | RAL 1019 Grey beige | RAL 7035 Light grey | Moderate | _ |
|  | ISP 3 | RAL 6010 Grass green | RAL 7045 Tele grey 1 | Abundant | _ |
|  | ISP 4 | RAL 7004 Signal grey | RAL 9002 Grey white | Good | _ |
|  | ISP 5 | RAL 3011 Brown red | RAL 9002 Grey white | poor | _ |
| Ac-464 | ISP 2 | RAL 5003 Saphire blue | RAL 9003 Signal white | Abundant | Brown grey |
|  | ISP 3 | RAL 7021 Black grey | RAL 7004 Signal grey | Very good | _ |
|  | ISP 4 | RAL 5008 Grey blue | RAL 7004 Signal grey | Good | _ |
|  | ISP 5 | RAL 1005 Honey yellow | RAL 9001 Cream | poor | _ |
| Go-466 | ISP 2 | RAL 7013 Brown grey | RAL 9006 White Aluminium | Moderate | _ |
|  | ISP 3 | RAL 7022 Umbra grey | RAL 1013 Oyster white | Excellent | Chocolate Brown |
|  | ISP 4 | RAL 1015 Light Ivory | RAL 8004 Cooper brown | Good | _ |
|  | ISP 5 | RAL 9002 Grey white | RAL 9007 Grey aluminum | poor | _ |
| Go-475 | ISP 2 | RAL 7013 Brown grey | RAL 9003 Signal white | Moderate | Green grey |
|  | ISP 3 | RAL 8022 Black brown | RAL 7035 Light grey | Excellent | Terra brown |
|  | ISP 4 | RAL 3004 Purple red | RAL 9016 Traffic white | Moderate | _ |
|  | ISP 5 | RAL 9002 Grey white | RAL 9018 Papyrus white | Moderate | _ |

**Table S4.** Important physiological and biochemical test results of the isolates.

| **Isolates** | **Test for** | | | | | | **Growth at various**  **NaClconc**. (%) | | | | | **pH**  **opt.** | **Temp.**  **opt.** |
| --- | --- | --- | --- | --- | --- | --- | --- | --- | --- | --- | --- | --- | --- |
|  | Starch hydrolysis | Chitinase | Oxidase | Catalase | Indole production | Melanin | 1.25 | 2.5 | 5 | 7.5 | 10 |  |  |
| Ac-006 | + | + | _ | + | _ | _ | + | + | + | ± | _ | 7.0 | 25 ^0^C |
| Ac-029 | + | + | _ | + | _ | + | + | + | + | + | ± | 7.5 | 30 ^0^C |
| Ed-065 | + | + | _ | + | _ | _ | + | + | + | + | ± | 7.5 | 30 ^0^C |
| Ac-123 | + | + | + | + | _ | + | + | + | + | + | ± | 7.5 | 30 ^0^C |
| Ac-125 | + | + | + | + | _ | _ | + | + | + | ± | _ | 7.0 | 25 ^0^C |
| Ac-146 | + | + | + | + | _ | + | + | + | + | ± | _ | 7.5 | 30 ^0^C |
| Rv-355 | + | + | + | + | _ | _ | + | + | + | + | ± | 7.5 | 30 ^0^C |
| Ac-464 | + | + | + | + | _ | + | + | + | + | ± | _ | 7.5 | 30 ^0^C |
| Go-466 | + | + | + | + | _ | _ | + | + | + | + | ± | 7.0 | 25 ^0^C |
| Go-475 | + | + | + | + | _ | _ | + | + | + | + | ± | 7.5 | 30 ^0^C |

+ test positive or the presence of growth, _ test negative or no growth, ± growth is very scarce or doubtful

**Table S5.** Proposed structure, retention time, HRMS data and predicted sum formulas of the compounds tentatively identified in the extracts from *Streptomyces* sp. Go-475 cultivated in liquid and solid media using high-resolution LC-MS.

| **#** | **Proposed structure** | **R_t_**  **[min]** | **[M+H]^+^ or [M+2H]^2+^** | | **Δm/z**  **[ppm]** | **MW exp.**  **[Da]** | **Predicted**  **sum formula** |
| --- | --- | --- | --- | --- | --- | --- | --- |
|  |  |  | *m/z* exp. | *m/z* calc. |  |  |  |
| 1 | Ectoine | 2.5^a^ | 143.0818 | 143.0815 | -2.3 | 142.0745 | C_6_H_10_N_2_O_2_ |
| 2 | Hydroxyectoine | 2.5^a^ | 159.0767 | 159.0764 | -1.7 | 158.0694 | C_6_H_10_N_2_O_3_ |
| 3 | Anthracycline antibiotic | 2.5^a^ | 591.2691 | 591.2674 | -2.9 | 1180.5236 | C_60_H_80_N_2_O_22_ |
| 4 | Anthracycline antibiotic | 2.5^a^ | 593.2841 | 593.2831 | -1.8 | 1184.5536 | C_60_H_84_N_2_O_22_ |
| 5 | Cosmomycin D | 2.5^a^ | 595.2988 | 595.2987 | -0.2 | 1188.5830 | C_60_H_88_N_2_O_22_ |
| 6 | Betaine | 2.6^a^ | 118.0867 | 118.0863 | -3.9 | 117.0794 | C_5_H_11_NO_2_ |
| 7 | Anthracycline antibiotic | 3.7 | 450.2313 | 450.2304 | -1.9 | 898.4480 | C_47_H_66_N_2_O_15_ |
| 8 | Anthracycline antibiotic | 3.7 | 457.2390 | 457.2383 | -1.6 | 912.4634 | C_48_H_68_N_2_O_15_ |
| 9 | Anthracycline antibiotic | 3.7 | 465.2366 | 465.2357 | -1.9 | 928.4586 | C_48_H_68_N_2_O_16_ |
| 10 | Anthracycline antibiotic | 3.7 | 473.2338 | 473.2332 | -1.3 | 944.4530 | C_48_H_68_N_2_O_17_ |
| 11 | Anthracycline antibiotic | 3.7 | 481.2312 | 481.2306 | -1.2 | 960.4478 | C_48_H_68_N_2_O_18_ |
| 12 | Cosmomycin A | 3.7 | 756.3581 | 756.3590 | 1.1 | 755.3508 | C_40_H_53_NO_13_ |
| 13 | Cosmomycin B | 3.7 | 772.3535 | 772.3539 | 0.5 | 771.3462 | C_40_H_53_NO_14_ |
| 14 | Anthracycline antibiotic | 3.7 | 788.3482 | 788.3488 | 0.8 | 787.3409 | C_40_H_53_NO_15_ |
| 15 | Anthracycline antibiotic | 3.7 | 802.3639 | 802.3644 | 0.7 | 801.3566 | C_41_H_55_NO_15_ |
| 16 | **8-O-Methyltetrangomycin** | 4.0 | 337.1081 | 337.1071 | -3.2 | 336.1008 | C_20_H_16_O_5_ |
| 17 | 4-Methoxy-1(3H)-isobenzofuranone^b^ | 4.9 | 165.0547 | 165.0546 | -0.5 | 164.0474 | C_9_H_8_O_3_ |
| 18 | 3-Phenylpropionic acid^b^ | 6.8 | 151.0755 | 151.0754 | -0.8 | 150.0682 | C_9_H_10_O_2_ |
| 19 | **8-O-Methyltetrangulol** | 13.7 | 319.0970 | 319.0965 | -1.7 | 318.0897 | C_20_H_14_O_4_ |
| 20 | Albaflavenone | 16.9 | 219.1748 | 219.1743 | -2.2 | 218.1675 | C_15_H_22_O |
| 21 | Dehydrocineromycin B^b^ | 22.3 | 293.1747 | 293.1747 | 0.3 | 292.1674 | C_17_H_24_O_4_ |

^a^ These compounds elute unretained in the solvent peak.

^b^ Not supported by genome analysis.

**Figure S1.** Map of Ethiopia (prepared by Samson Warkaye Lemma and Moges Kibret), where the 13 specific soil sample collection sites are indicated in black triangles, arrows signify the sampling points for the six selected isolates for further investigation.


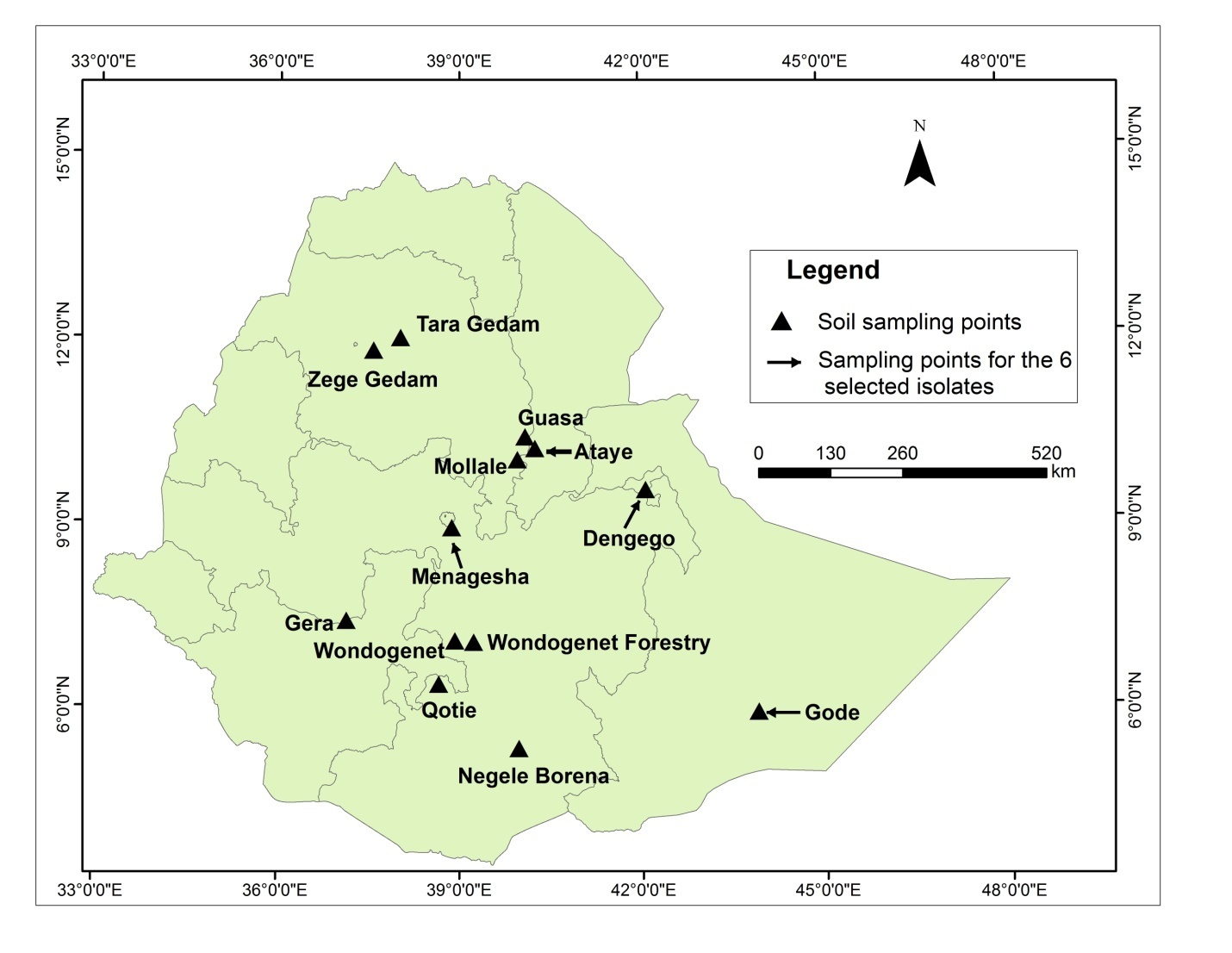


Collection point **Ataye** has an elevation of 1455 m above sea level around the transition between middle and lower Awash river basin, which is part of the great rift valley of Ethiopia. The area is semi-arid, characterized by scant vegetation, dark colored alluvial soils with medium clay content, low in lime content and fine in textures. Another sampling site is **Dengego** in the eastern geographical region in Ethiopia located at an altitude of 2111 m. The area is relatively arid, with most of the rain falling during the short rainy season (March to April) and the long rainy season (July to September). The area is very thin to non-residual soils on high to mountainous relief parallel ridges of Precambrian granites and limestone. Sampling site namely **Gode** is located in a low land area with an elevation of 295 m above sea level. The climate is equatorial, mostly characterized as desert ecosystem with yellowish–brown sandy soil. Gode is an arid land marked by drought, seasonal variations and the mean annual rainfall about 200 mm. **Menagesha Suba State Forest** is one of the ancient forests in Ethiopia, represented by an isolated forest block located in central Ethiopia. The area is reasonably well conserved volcanic dome of mountain Wachacha, with light brown to reddish brown soils. The mean annual rainfall at Menagesha forest has been estimated to be 1314 mm, and it is the home of different wild animals including 108 species of birds out of which seven are endemics to Ethiopia.

**Figure S2**. Genome synteny of *Streptomyces* sp. Go-475 and *S. olindensis* DAUFPE 5622.


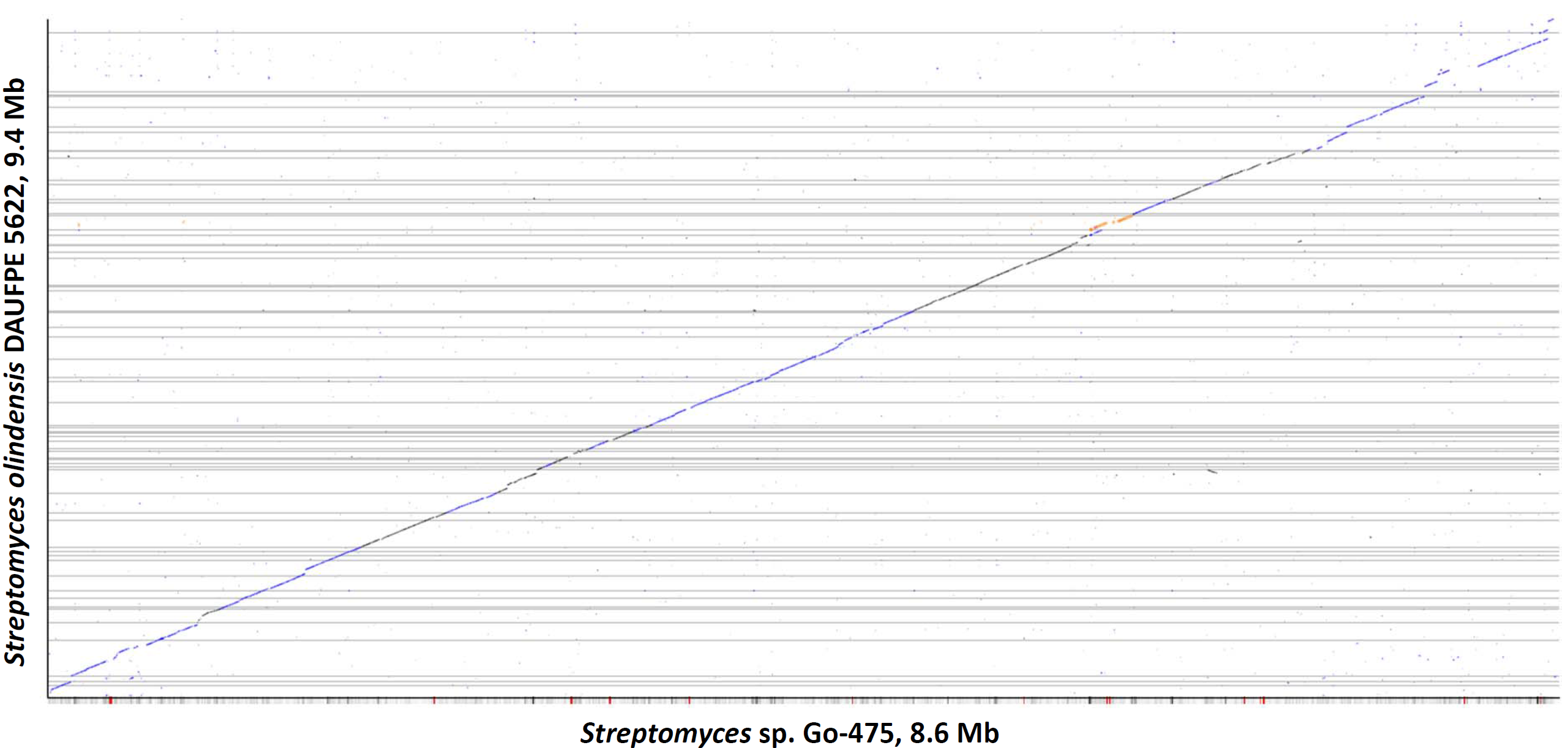


**Figure S3.** MS/MS spectrum of the [M+H]^+^ ion of 8-O-Methyltetrangomycin (6-deoxy-8-O-methylrabelomycin) at *m/z* 337.1074 obtained on an ESI-Qq-TOF mass spectrometer with a collision energy of 25 eV.

**Figure S4.** MS/MS spectrum of the [M+H]^+^ ion of 8-O-Methyltetrangomycin (6-deoxy-8-O-methylrabelomycin) at *m/z* 337.1074 obtained on an ESI-Qq-TOF mass spectrometer with a collision energy of 40 eV.

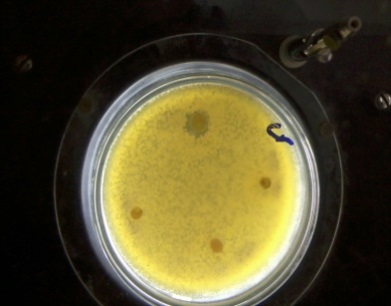
**Figure S5.** Inhibition of some test organisms by the extracts from selected *Streptomyces* spp.


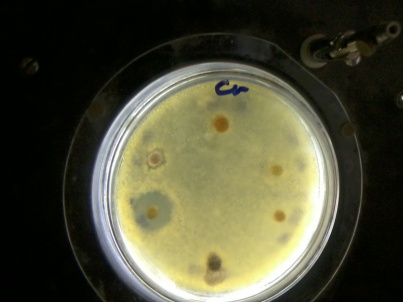
 A

Go-475 against *C.neoformans* Ac-006 against *C.neoformans*


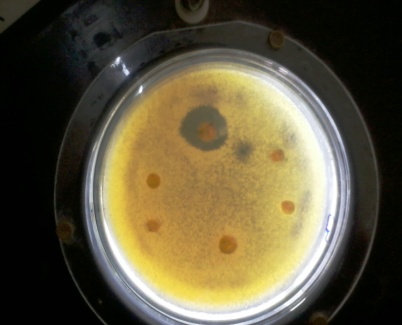

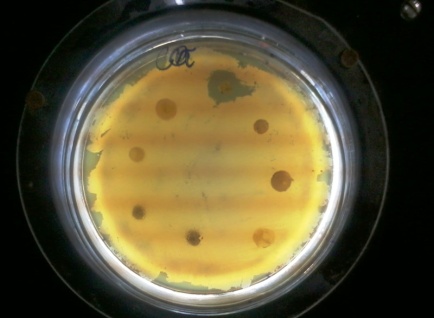


Ac-006 against *C.albicans* Ru-355 againest *C.neoformans*


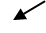

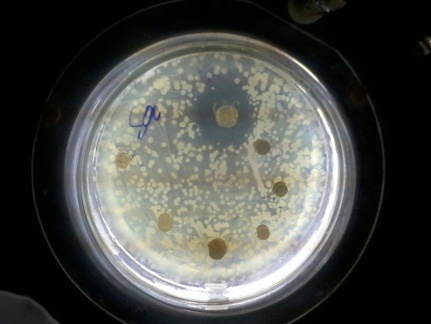

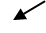

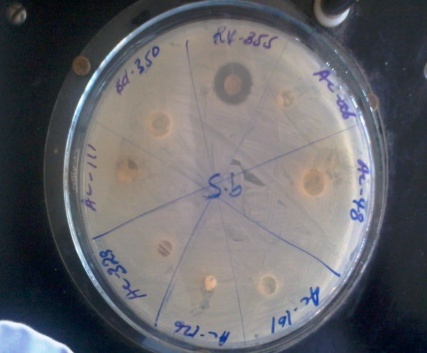


Ru-355 against *Shigella boydii* Ed-065 against *S. aureus*


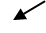

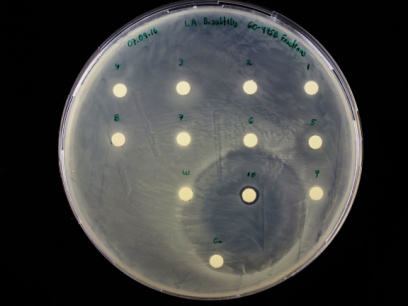


Go-475 against *B. subtilis*

**Description of the clinical isolates used as test organisms in antimicrobial assays**

*Cryptococcus neoformans* clinical isolate was initially evaluated and identified by standard mycological techniques, based on the growth appearance on Sabouraud’s dextrose agar (SDA) at 37 ^o^C, and the presence of a capsule in microscopic observation using India ink preparation. Microscopic observation of the culture showed the presence of rounded and capsulated yeast cell with India ink. It showed mucoid, soft in texture and creamy-colored colonies that have been turned in to tan or brown on prolonged incubation. In repeated subculturing it showed colonies with dry surface appearance.

The *Shigella boydii* clinical isolate was obtained from Microbiology laboratory stock culture. It showed well growth at 37^o^C on nutrient agar and Muller Hinton agar media.

**Chemical shifts recorded for purified compounds during NMR-based structure elucidation.**

3,4-Dihydro-3-hydroxy-8-methoxy-3-methyl-benz[a]anthracene-1,7,12(2H)-trione
= 8-O-methyltetrangomycin (**1**):

^1^H (500 MHz, d_4_-Methanol) δ = 8.26 (d, 8.1 Hz, 1H, H-6), 7.81 (dd, 8.5 and 7,1 Hz, 1H, H-10), 7.67 (d, 7,1 Hz, 1H, H-11), 7.66 (d, 8,1 Hz, 1H, H-5), 7.51 (d, 8.5 Hz, 1H, H-9), 4.01 (s, 3H, OMe), 3.26 (d, 16.9 Hz, 1H, H-4/1), 3.15 (d, 16.9 Hz, 1H, H-4/2), 3.09 (d, 14.3 Hz, 1H, H-2/1), 2.88 (d, 14.3 Hz, 1H, H-2/2), 1.45 (s, 3H, CH_3_).

^13^C (125 MHz, d_4_-Methanol) δ = 199.36 (C-1), 186.04 (C-12), 182.88 (C-7), 161.33 (C-8), 149.24 (C-4a), 139.02 (C-11a), 137.02 (C-10), 136.48 (C-12a), 136.11 (C-6a), 135.44 (C-12b), 135.30 (C-5), 130.70 (C-6), 121.43 (C-7a), 120.14 (C-11), 118.90 (C-9), 73.21 (C-3), 56.89 (OMe), 54.27 (C-2), 44.64 (C-4), 29.84 (CH_3_).

1-Hydroxy-8-methoxy-3-methyl-benz[a]anthracene-7,12-dione
= 8-O-methyltetrangulol (**2**)**:**

^1^H (500 MHz, CDCl_3_) δ = 11.15 (S, 1H, OH), 8.30 (d, 8.7 Hz, 1H, H-6), 8.13 (d, 8.7 Hz, 1H,
H-5), 7.95 (d, 8.5 Hz, 1H, H-11), 7.75 (dd, 8.5 and 7.8 Hz, 1H, H-10), 7.36 (d, 7.8 Hz, 1H, H-9), 7.24 (s, 1H, H-4), 7.14 (s, 1H, H-2), 4.08 (s, 3H, OMe), 2.49 (s, 3H, CH_3_).

^13^C (125 MHz, CDCl_3_) δ = 190.77 (C-12), 182.25 (C-7), 159.51 (C-8), 154.98 (C-1), 141.16
(C-3), 138.33 (C-12a), 137.58 (C-5), 137.28 (C-11a), 136.69 (C-6a), 135.30 (C-10), 130.67
(C-4a), 122.76 (C-6), 121.16 (C-4), 120.98 (C-11), 119.82 (C-7a), 119.71 (C-2), 119.12 (C-12b), 118.15 (C-9), 56.62 (OMe), 21.24 (CH_3_).
